# Supplementary figures and images for: RAF1 as a standalone therapeutic target in KRAS-driven lung adenocarcinoma: No added efficacy from co-targeting ARAF, EGFR, or DDR1
Source: PLoS One. 2026 Feb 6;21(2):e0341778. doi: 10.1371/journal.pone.0341778 (PMC12880662; doi:10.1371/journal.pone.0341778)

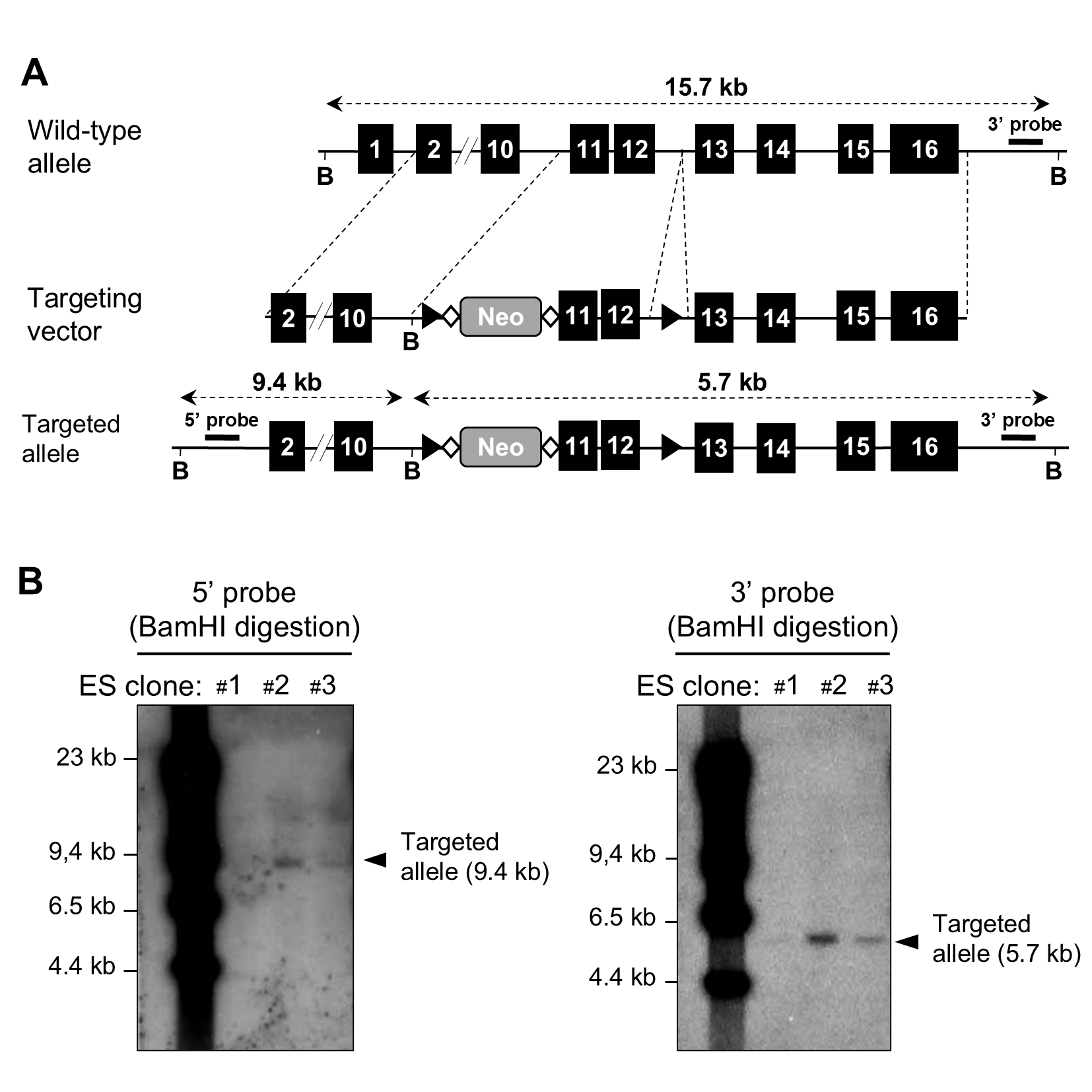

Supplement: S1 Fig — (A) Schematic representation of the wild-type Araf locus, the targeting vector and the targeted allele. Black boxes, Araf exons; Neo, PGK-Neomycine cassette; white diamond, FRT site; black triangle, loxP site. The positions of the probes (5’ and 3’), and restriction enzyme sites (B, BamHI) are indicated. The expected diagnostic fragments following BamHI digestion are represented by arrows. (B) Southern blot analysis of gDNA from recombinant ES cell clones carrying the recombinant Araf allele. (TIF) [file pone.0341778.s001.tif]

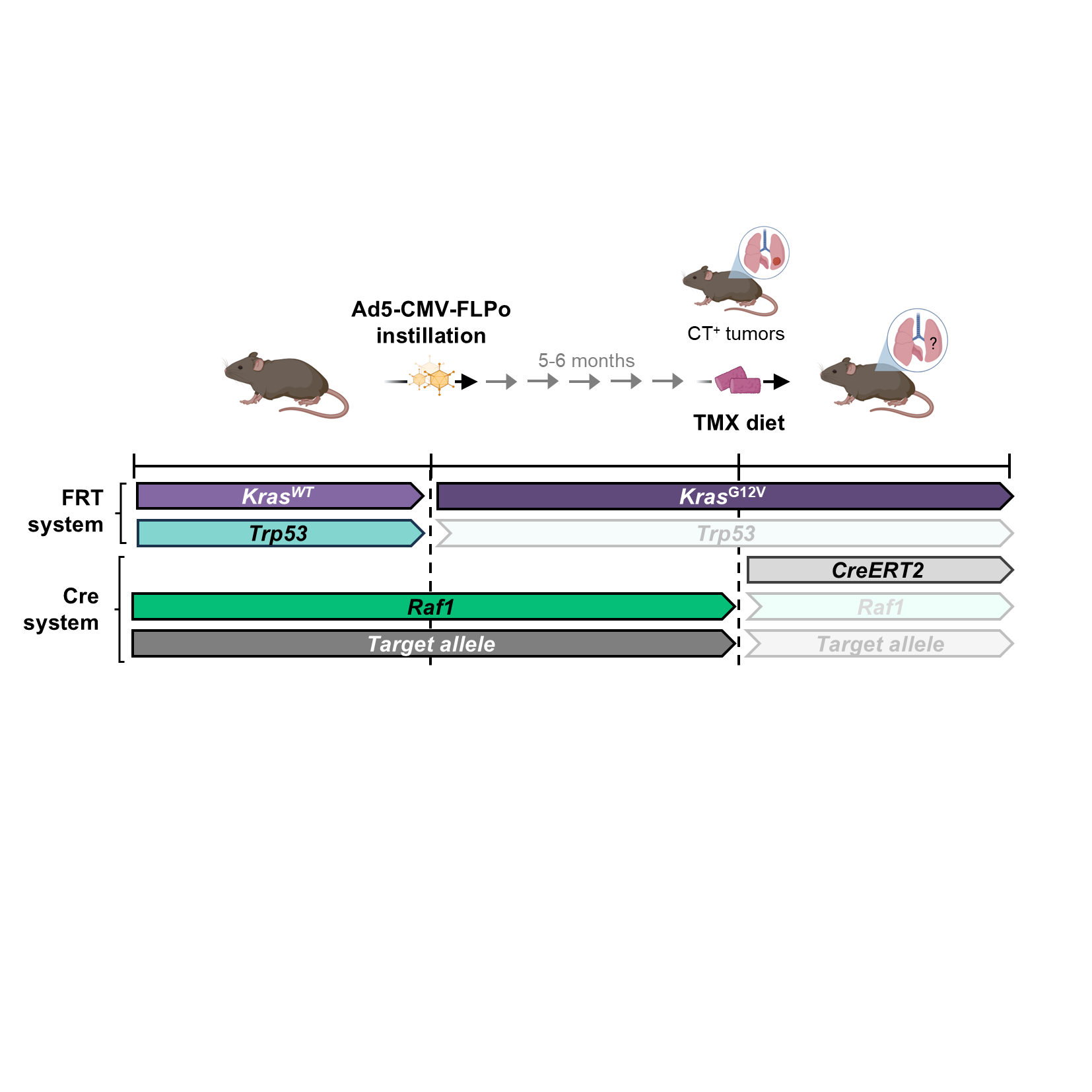

Supplement: S2 Fig — Schematic representation of gene expression and ablation during lung adenocarcinoma tumor development. The KrasFSFG12V and Trp53F alleles undergo recombination following Ad5-CMV-FLPo infection. The hUBC-CreERT2T, Raf1L, and the additional targeted alleles are either activated or recombined upon exposure to TMX. Dark colored boxes represent gene expression, whereas light colored boxes correspond to ablated genes. (TIF) [file pone.0341778.s002.tif]

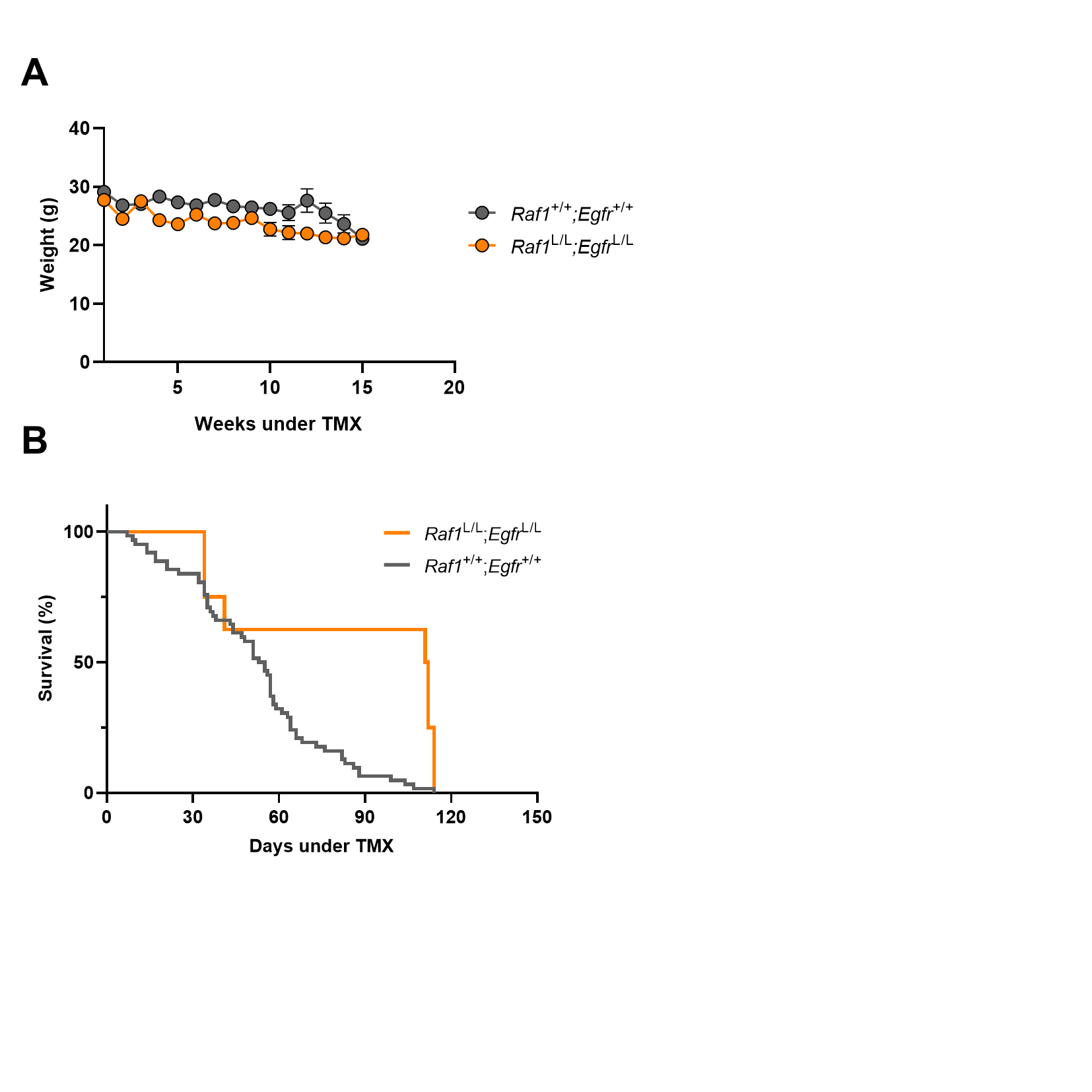

Supplement: S3 Fig — (A) Body weight measurements (in grams) of mice exposed to TMX-diet over time. Each point represents the mean weight of a group of mice, with error bars indicating the standard error of the mean (SEM). The gray points represent Raf1+/+;Egfr+/+ mice (n = 60), while the orange points represent Raf1L/L;EgfrL/L mice (n = 19). (B) Kaplan-Meier survival curves representing data from Raf1+/+;Egfr+/+ (n = 62, grey), Raf1L/L;EgfrL/L (n = 8, orange) mice. (TIF) [file pone.0341778.s003.tif]

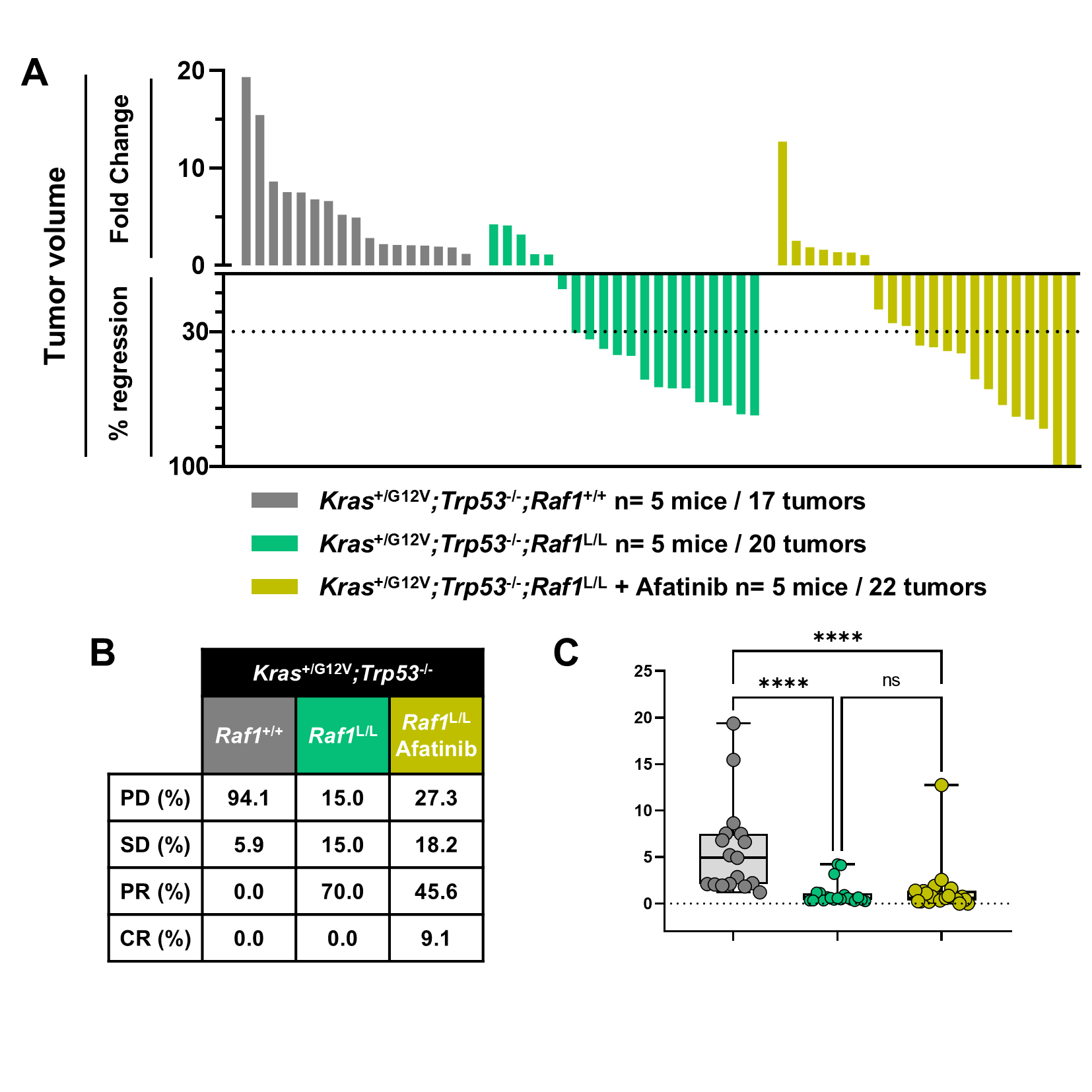

Supplement: S4 Fig — (A) Waterfall plot representing the FC in tumor volume and the percentage of regression for individual CT-positive lung tumors in Kras+/FSFG12V;Trp53F/F;hUBC-CreERT2+/T;Raf1+/+ (grey), Kras+/FSFG12V;Trp53F/F;hUBC-CreERT2+/T;Raf1L/L (green), and Kras+/FSFG12V;Trp53F/F;hUBC-CreERT2+/T;Raf1L/L + afatinib (mustard) mice following 2 months of treatment. (B) Table indicating the percentage of tumors that show PR, CR, PD, and SD. (C) Statistical comparison of tumor volume FC between groups. p-values were obtained using the Kruskal-Wallis test with multiple comparisons. Data are shown as in Fig 3. (TIF) [file pone.0341778.s004.tif]

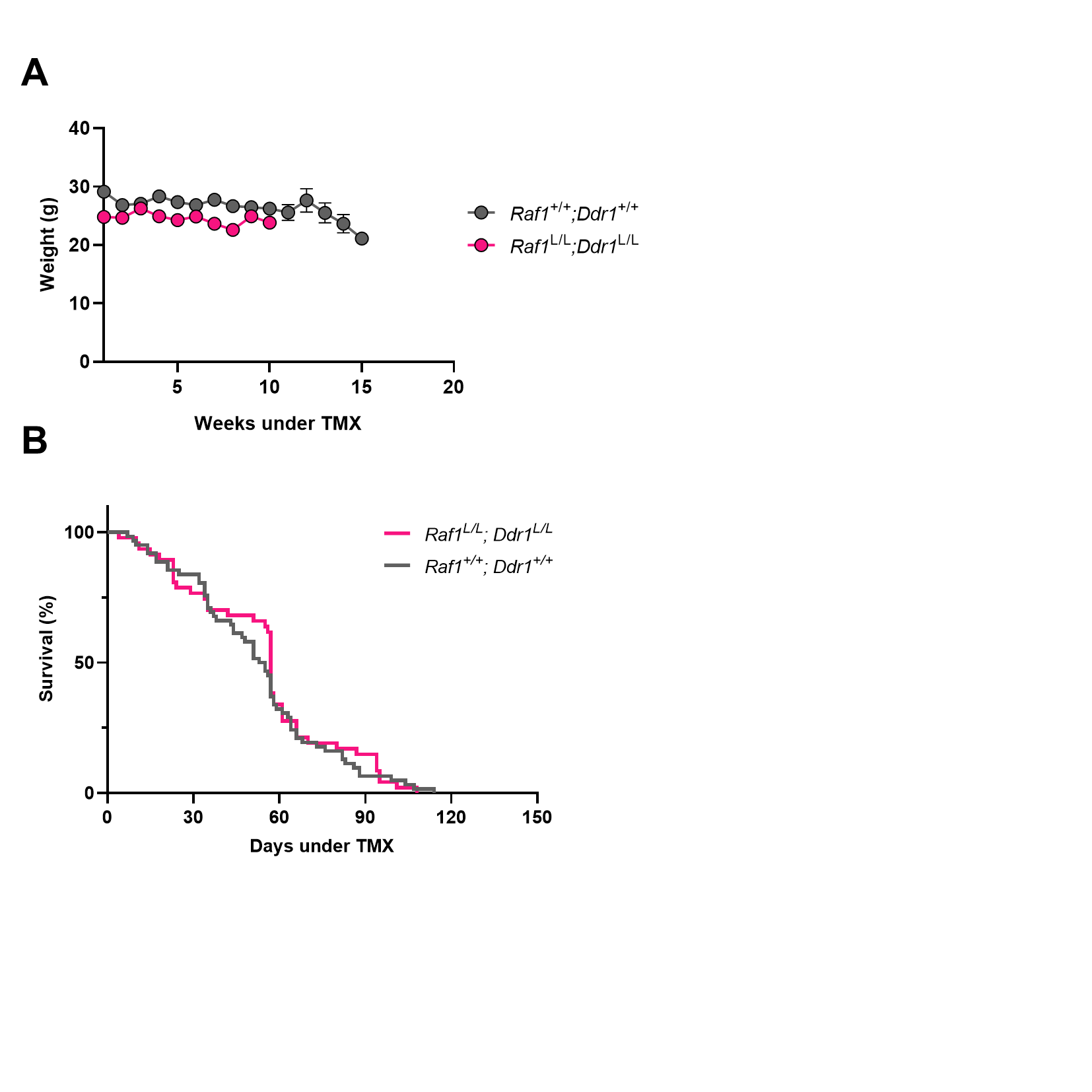

Supplement: S5 Fig — (A) Body weight measurements (in grams) of mice exposed to TMX-diet over time. Each point represents the mean weight of a group of mice, with error bars indicating the standard error of the mean (SEM). The gray points represent Raf1+/+;Ddr1+/+ mice (n = 60), while the pink points represent Raf1L/L;Ddr1L/L mice (n = 36). (B) Kaplan-Meier survival curves representing data from Raf1+/+;Ddr1+/+ (n = 62, grey), Raf1L/L;Ddr1L/L (n = 47, pink) mice. (TIF) [file pone.0341778.s005.tif]

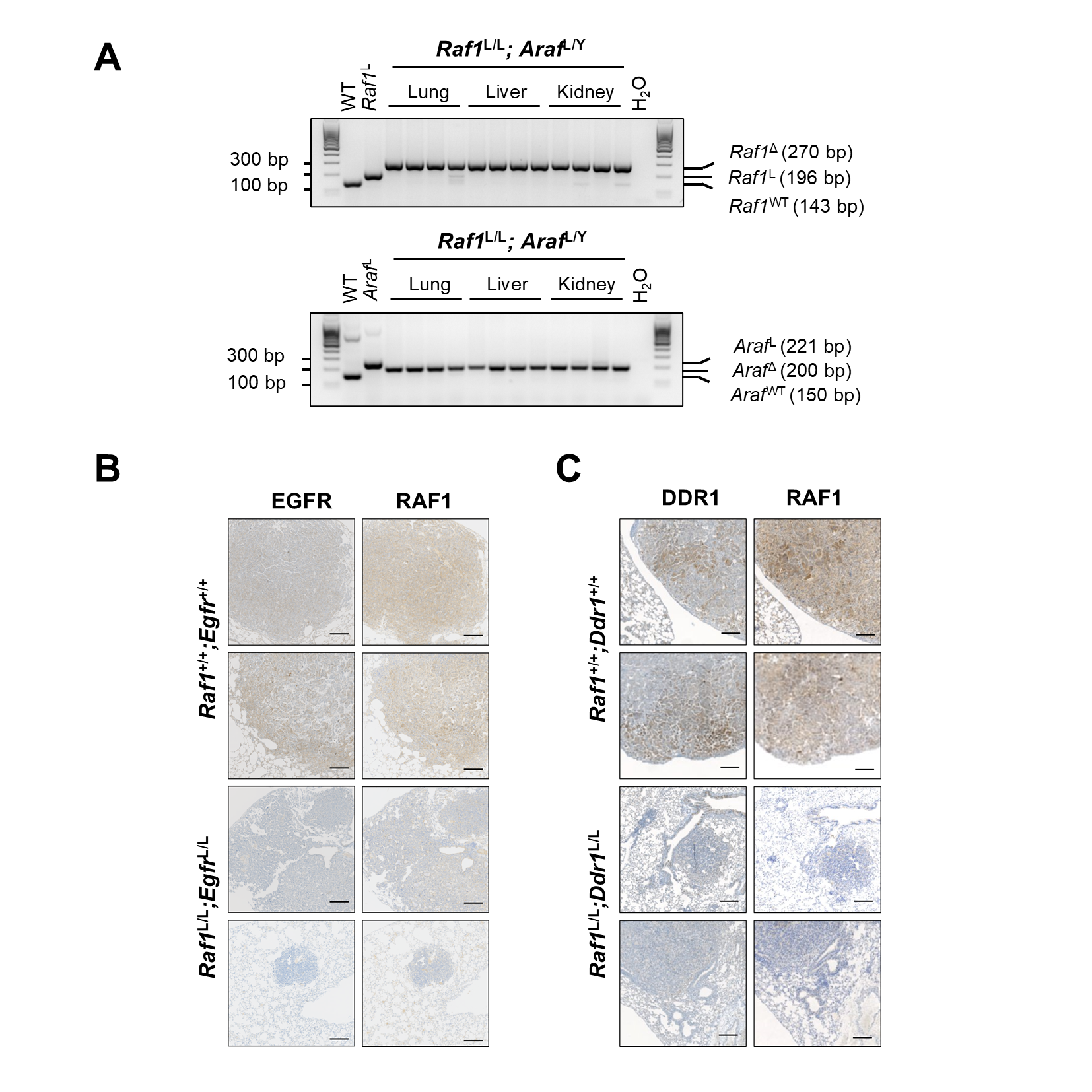

Supplement: S6 Fig — (A) Systemic Raf1 and Araf ablation confirmed in representative tissues. Upper panel, PCR analysis of Raf1 ablation; lower panel, PCR analysis of Araf ablation. Lanes include WT, lox control, experimental samples, and a water negative control. (B–C) Representative immunostaining of paraffin-embedded lung tumor sections showing α-RAF1 in combination with α-EGFR (B) or α-DDR1 (C). Scale bars: 200 μm. (TIF) [file pone.0341778.s006.tif]

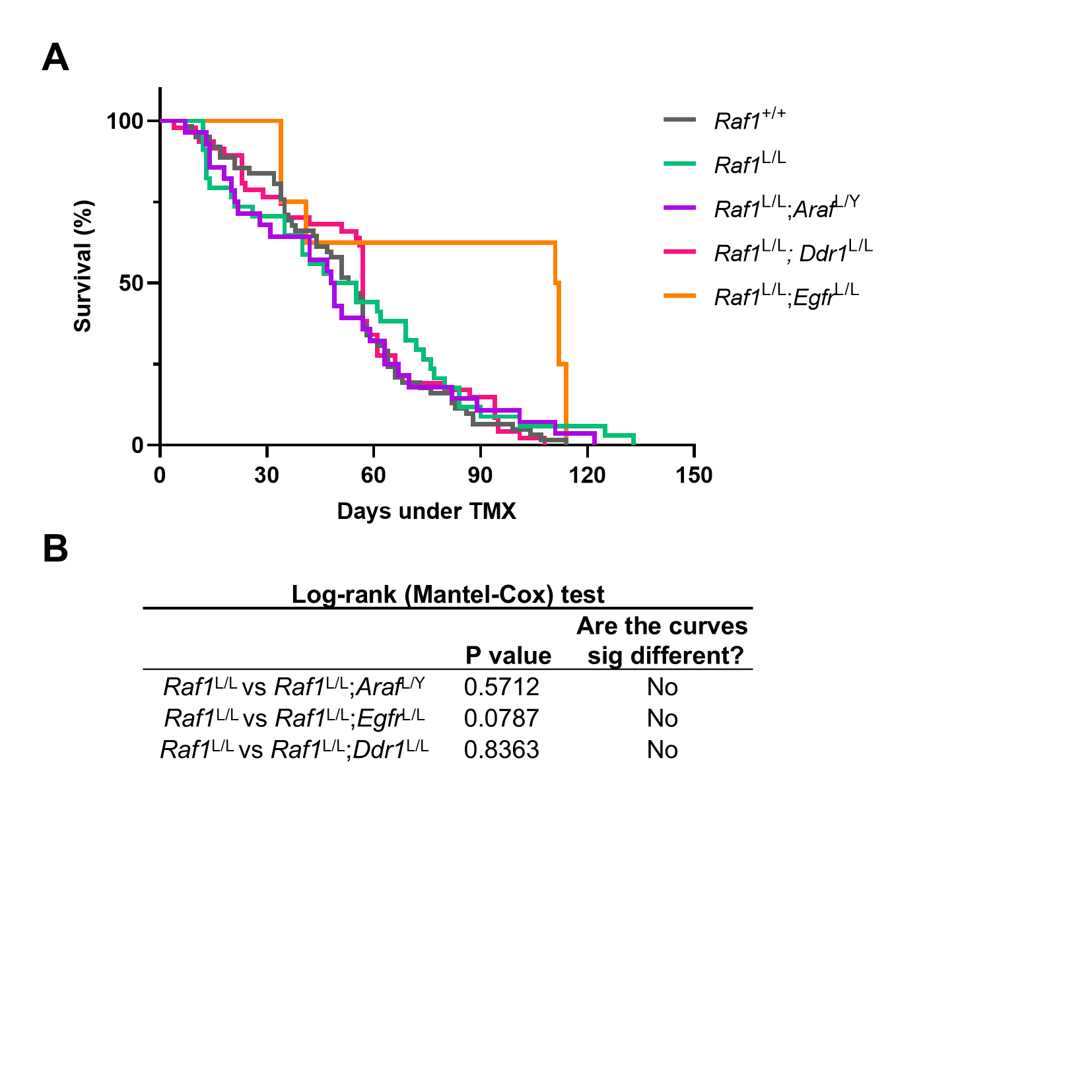

Supplement: S7 Fig — (A) Survival curves representing data from wild-type Raf1+/+ (n = 62, grey), Raf1L/L (n = 34, green), Raf1L/L;ArafL/Y (n = 28, purple), Raf1L/L;Ddr1L/L (n = 47, pink), Raf1L/L;EgfrL/L (n = 8, orange) mice. (B) Log-rank (Mantel–Cox) test comparing Raf1 single ablation with Raf1 ablation in combination with a second target. (TIF) [file pone.0341778.s007.tif]
