# Supplementary material for: RAF1 as a standalone therapeutic target in KRAS-driven lung adenocarcinoma: No added efficacy from co-targeting ARAF, EGFR, or DDR1
Source: PLoS One. 2026 Feb 6;21(2):e0341778. doi: 10.1371/journal.pone.0341778 (PMC12880662; doi:10.1371/journal.pone.0341778)
Supplement: S1 Table — Capital ‘L’, ‘F’, and ‘T’ stand for ‘loxP’, ‘FRT’, or ‘transgene’ respectively. (DOCX) [file pone.0341778.s008.docx]

**S1 Table. Description of the modified alleles present in the compound mouse strains.** Capital ‘L’, ‘F’, and ‘T’ stand for ‘loxP’, ‘FRT’ or ‘transgene’ respectively.

| **Allele** | **Description** |
| --- | --- |
| *Araf*^L^ | Exons 11-12 in the *Araf* gene are flanked by loxP sites. Exposure to Cre recombinase removes the floxed sequence, creating a null allele. |
| *Ddr1*^L^ | Exons 5-6 in the *Ddr1* gene are flanked by loxP sites. Exposure to Cre recombinase removes the floxed sequence, creating a null allele. |
| *Egfr*^L^ | Exon 1 in the *Egfr* gene are flanked by loxP sites. Exposure to Cre recombinase removes the floxed sequence, creating a null allele. |
| *Raf1*^L^ | Exon 3 in the *Raf1* gene is flanked by loxP sites. Exposure to Cre recombinase removes the floxed sequence, creating a null allele. |
| *Kras*^FSFG12V^ | A STOP cassette flanked by FRT sites followed by the G12V mutation is inserted into the exon 1 of *Kras* gene. Exposure to FLPo recombinase removes the STOP cassette allowing the KRAS^G12V^ oncoprotein expression. |
| *Kras*^LSLG12Vgeo^ | This is a modification of *Kras^LSLG12V^* allele, used specifically for the tumour initiation experiments. A STOP cassette followed by the G12V mutation in exon 1 and IRES-β-geo cassette is inserted into the *Kras* gene. Exposure to Cre recombinase removes the STOP cassette allowing the KRAS^G12V^ oncoprotein expression along with the β-galactosidase-NeoR (β-geo) fusion protein, which has lacZ activity that is used as a reporter. |
| *Trp53*^F^ | Exons 2-6 in the *Trp53* gene are flanked by FRT sites. Exposure to FLPo recombinase removes the flanked sequence, creating a null allele. |
| *Tg.hUBC-CreERT2*^T^ | Transgenic allele where Cre recombinase, under the control of the human ubiquitin C (hUBC) promoter, is fused to a modified oestrogen receptor that is selectively activated only in the presence of tamoxifen (TMX). |
