# Supplementary material for: RAF1 as a standalone therapeutic target in KRAS-driven lung adenocarcinoma: No added efficacy from co-targeting ARAF, EGFR, or DDR1
Source: PLoS One. 2026 Feb 6;21(2):e0341778. doi: 10.1371/journal.pone.0341778 (PMC12880662; doi:10.1371/journal.pone.0341778)
Supplement: S1 Raw Figures — Each object contains the underlying source data and statistical analyses, allowing for full transparency and replication of the experimental results. (PPTX) [file pone.0341778.s010.pptx]

## Slide 1
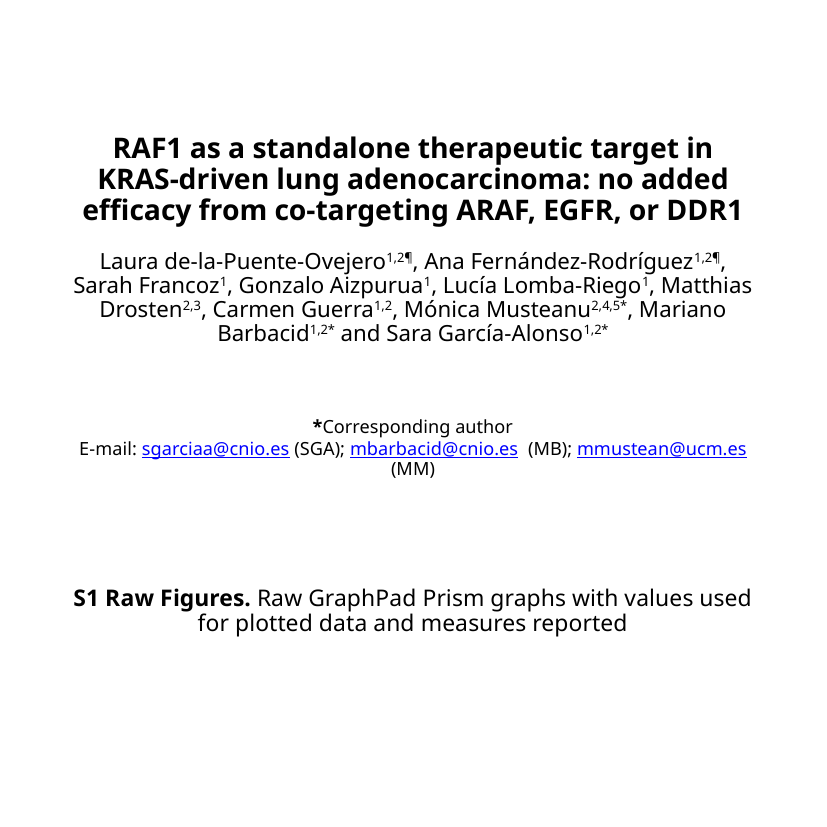

# RAF1 as a standalone therapeutic target in KRAS-driven lung adenocarcinoma: no added efficacy from co-targeting ARAF, EGFR, or DDR1 Laura de-la-Puente-Ovejero1,2¶, Ana Fernández-Rodríguez1,2¶, Sarah Francoz1, Gonzalo Aizpurua1, Lucía Lomba-Riego1, Matthias Drosten2,3, Carmen Guerra1,2, Mónica Musteanu2,4,5*, Mariano Barbacid1,2* and Sara García-Alonso1,2* *Corresponding author E-mail: sgarciaa@cnio.es (SGA); mbarbacid@cnio.es (MB); mmustean@ucm.es (MM)
S1 Raw Figures. Raw GraphPad Prism graphs with values used for plotted data and measures reported

## Slide 2
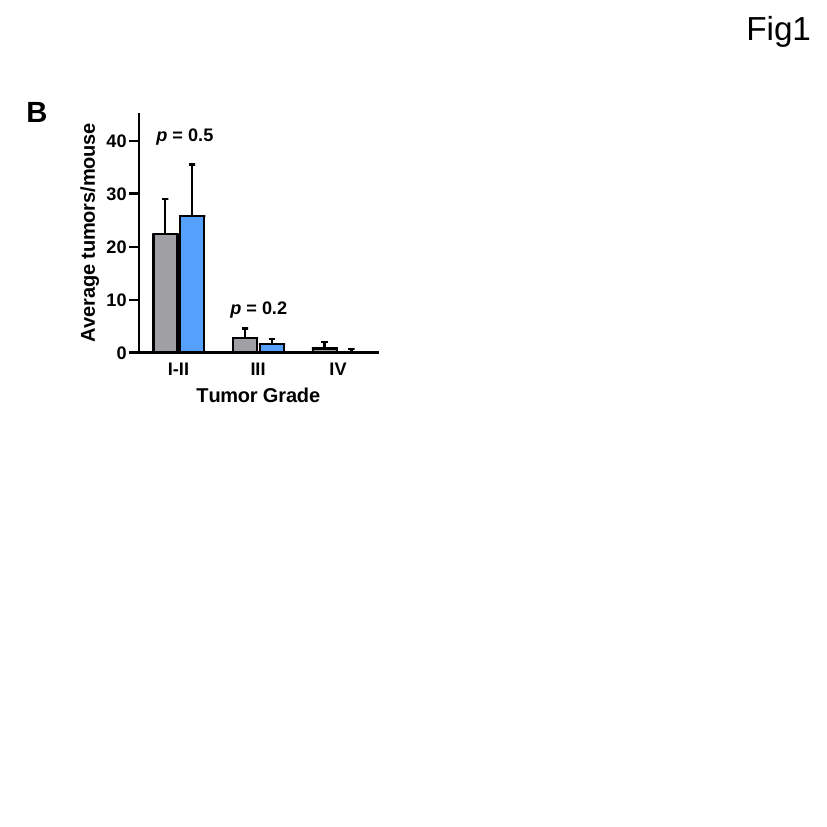

Fig1
B

## Slide 3
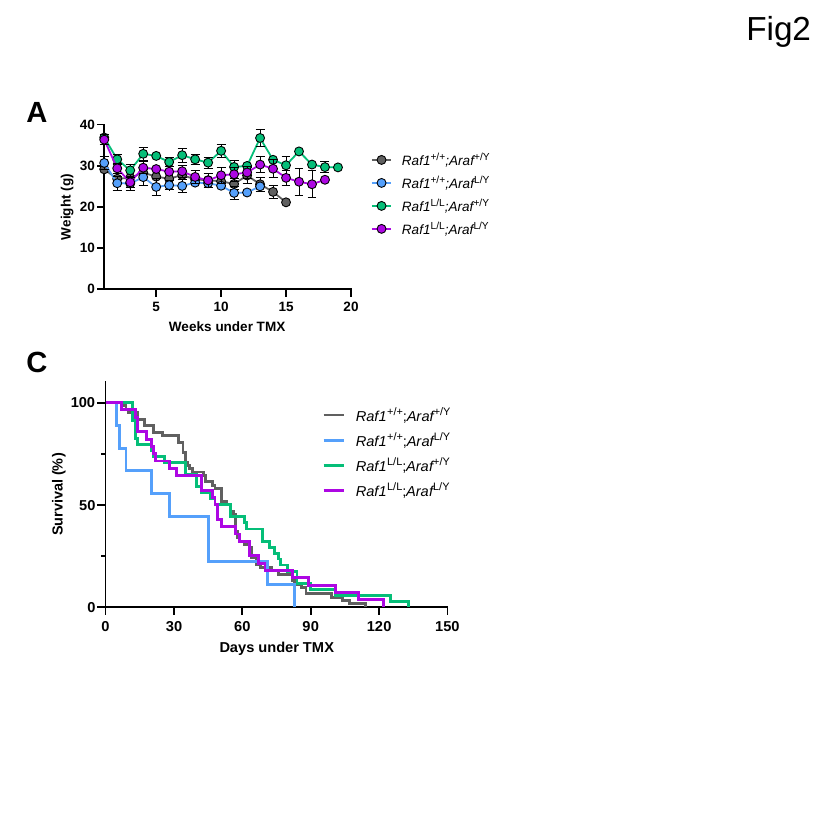

Fig2
A
C

## Slide 4
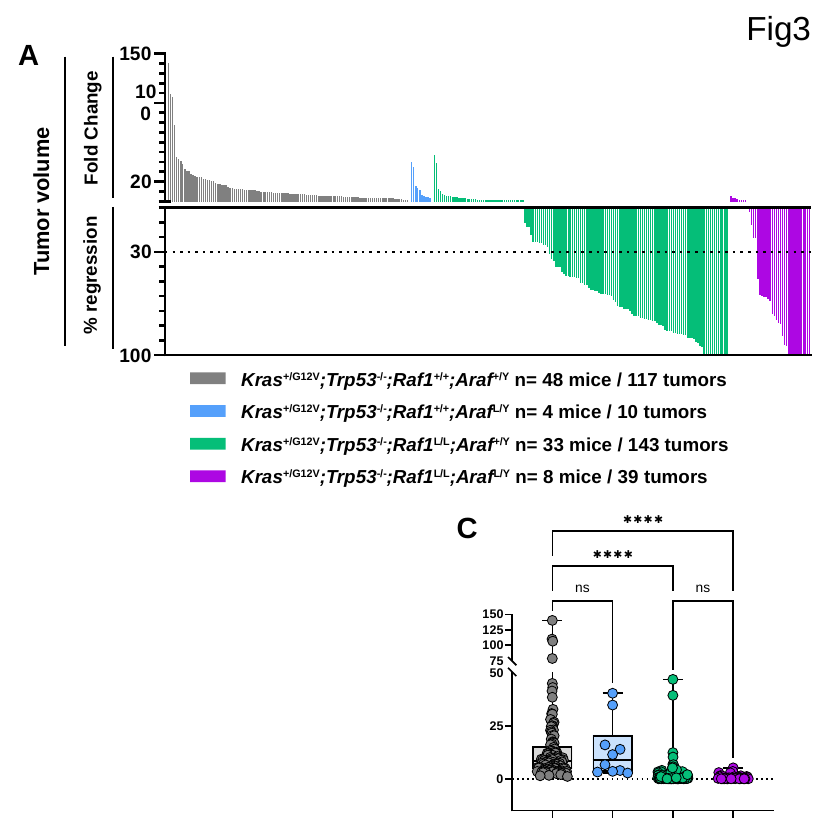

Fig3
Fold Change
Tumor volume
% regression
A
Kras+/G12V;Trp53-/-;Raf1+/+;Araf+/Y n= 48 mice / 117 tumors
Kras+/G12V;Trp53-/-;Raf1+/+;ArafL/Y n= 4 mice / 10 tumors
Kras+/G12V;Trp53-/-;Raf1L/L;Araf+/Y n= 33 mice / 143 tumors
Kras+/G12V;Trp53-/-;Raf1L/L;ArafL/Y n= 8 mice / 39 tumors
C

## Slide 5
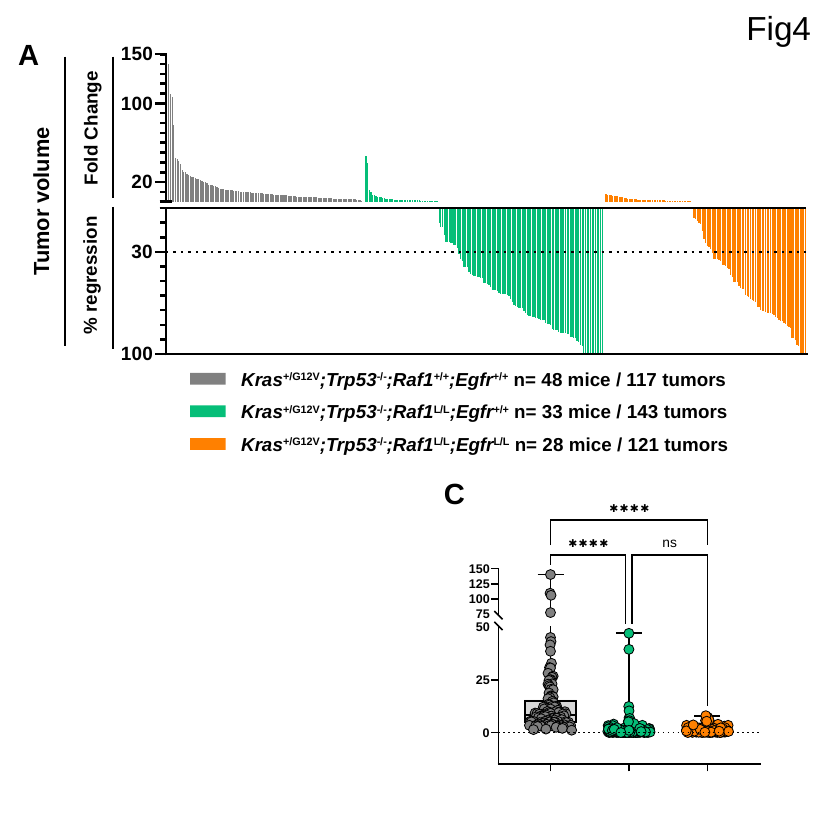

Fig4
Fold Change
Tumor volume
% regression
A
Kras+/G12V;Trp53-/-;Raf1+/+;Egfr+/+ n= 48 mice / 117 tumors
Kras+/G12V;Trp53-/-;Raf1L/L;Egfr+/+ n= 33 mice / 143 tumors
Kras+/G12V;Trp53-/-;Raf1L/L;EgfrL/L n= 28 mice / 121 tumors
C

## Slide 6
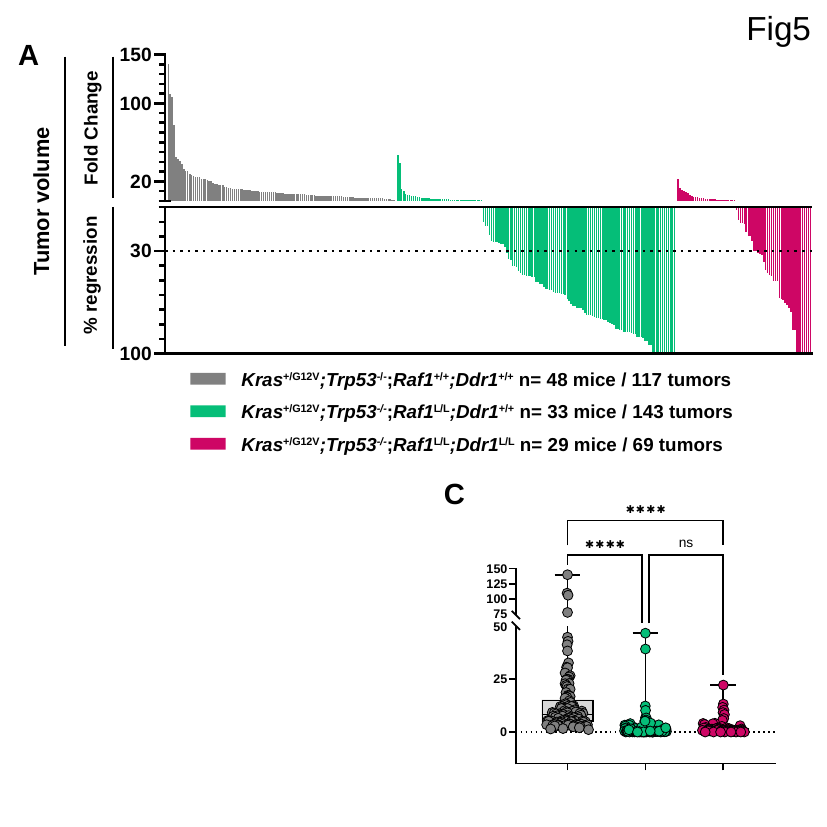

Fig5
Fold Change
Tumor volume
% regression
A
Kras+/G12V;Trp53-/-;Raf1+/+;Ddr1+/+ n= 48 mice / 117 tumors
Kras+/G12V;Trp53-/-;Raf1L/L;Ddr1+/+ n= 33 mice / 143 tumors
Kras+/G12V;Trp53-/-;Raf1L/L;Ddr1L/L n= 29 mice / 69 tumors
C

## Slide 7
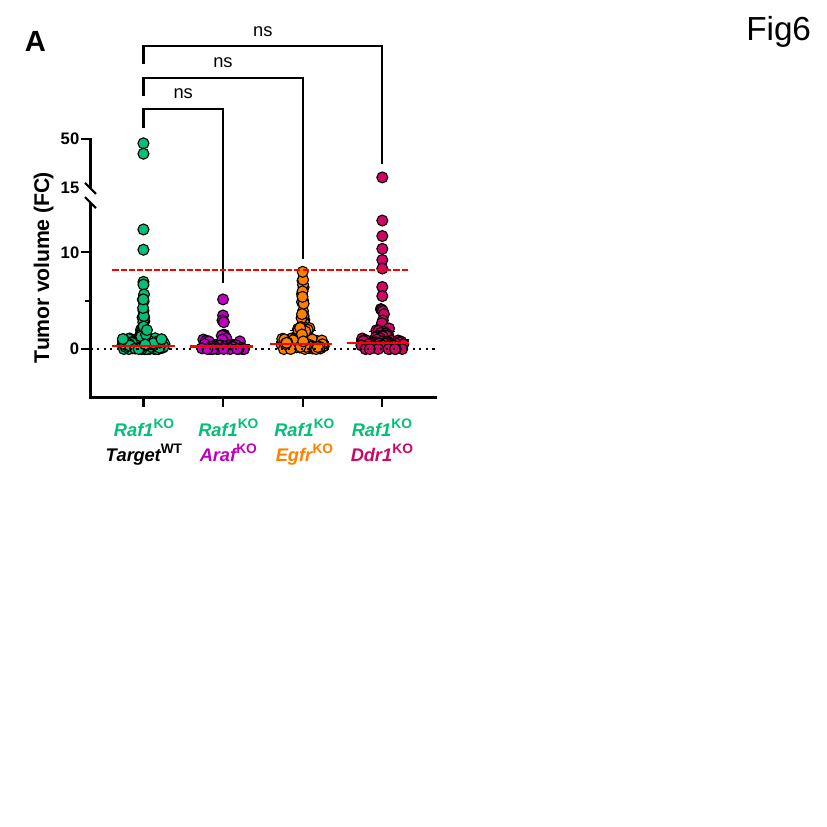

Fig6
A

## Slide 8
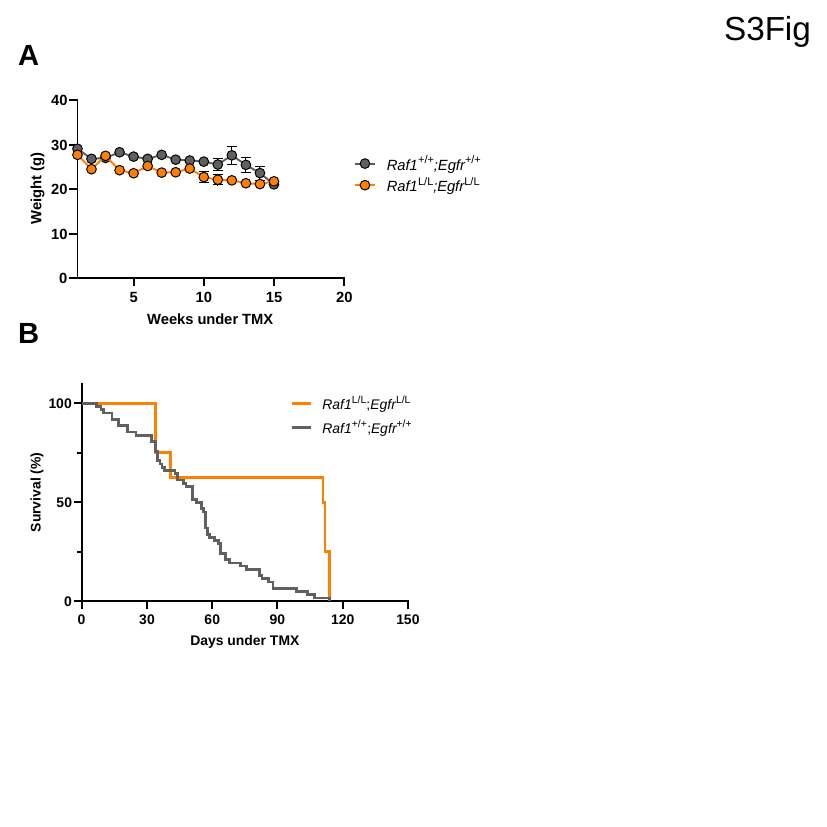

S3Fig
A
B

## Slide 9
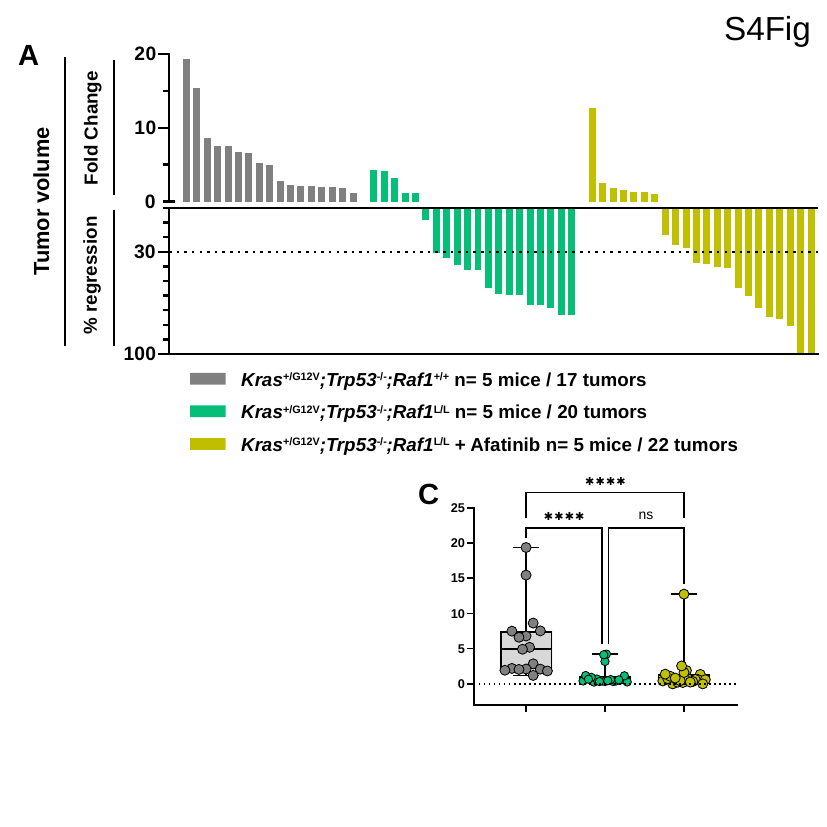

S4Fig
Fold Change
Tumor volume
% regression
A
Kras+/G12V;Trp53-/-;Raf1+/+ n= 5 mice / 17 tumors
Kras+/G12V;Trp53-/-;Raf1L/L n= 5 mice / 20 tumors
Kras+/G12V;Trp53-/-;Raf1L/L + Afatinib n= 5 mice / 22 tumors
C

## Slide 10
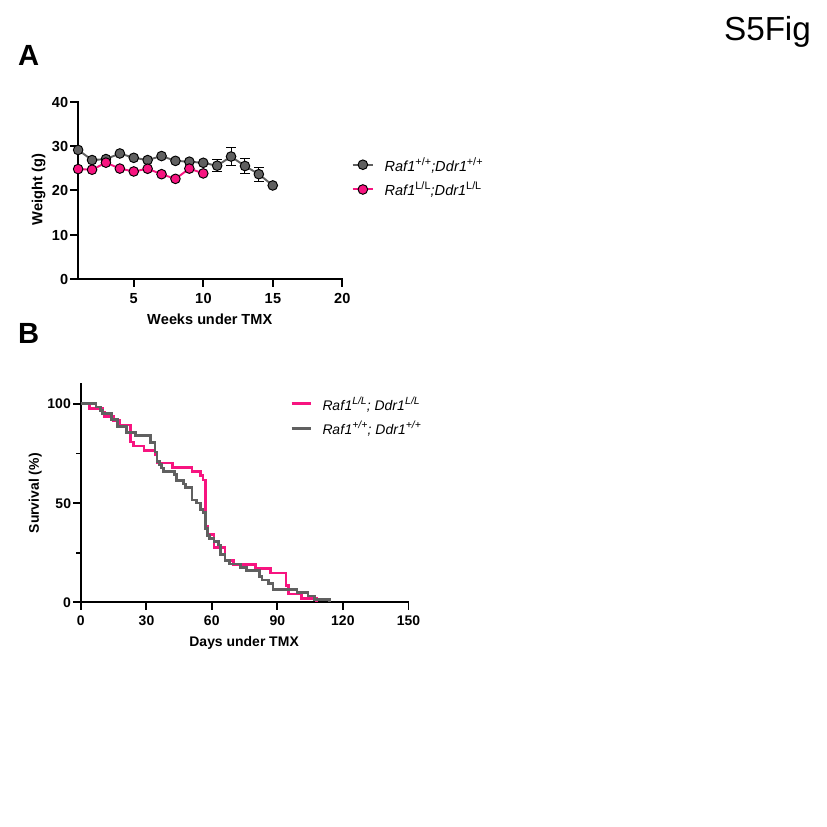

S5Fig
A
B

## Slide 11
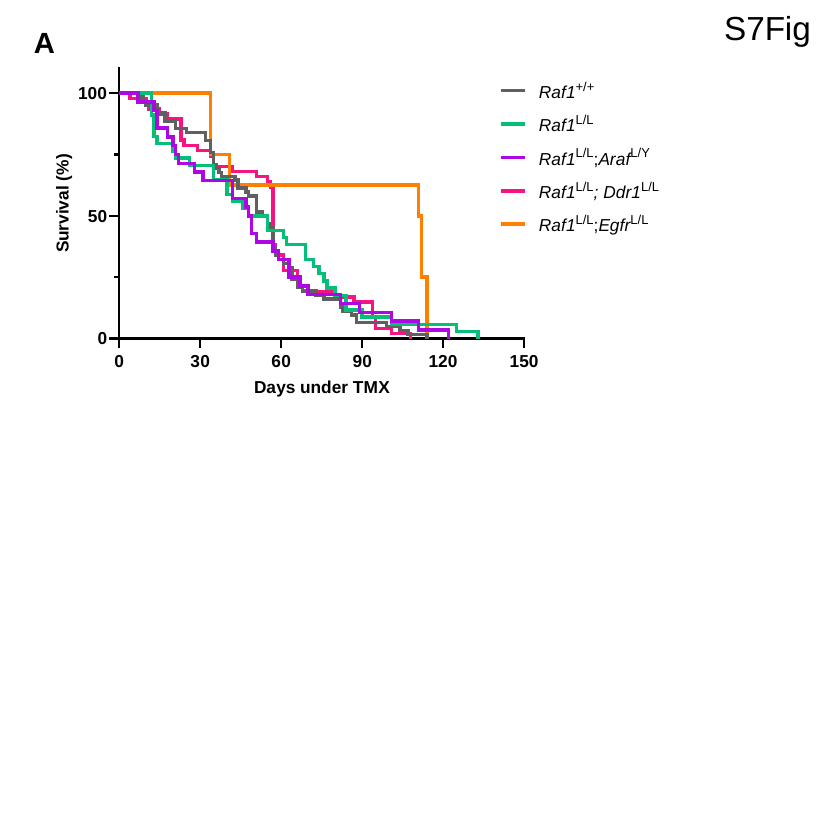

S7Fig
A
